# Rare central venous catheter position due to absence of the left brachiocephalic vein resembling lung penetration

**DOI:** 10.1093/omcr/omae063

**Published:** 2024-06-07

**Authors:** Haruka Taira, Masahiro Kashiura, Takashi Moriya

**Affiliations:** Department of Emergency and Critical Care Medicine, Saitama Medical Center, Jichi Medical University, 1-847 Amanuma-cho, Omiya-ku, Saitama-shi, Saitama 330-8503, Japan; Department of Emergency and Critical Care Medicine, Saitama Medical Center, Jichi Medical University, 1-847 Amanuma-cho, Omiya-ku, Saitama-shi, Saitama 330-8503, Japan; Department of Emergency and Critical Care Medicine, Saitama Medical Center, Jichi Medical University, 1-847 Amanuma-cho, Omiya-ku, Saitama-shi, Saitama 330-8503, Japan

**Keywords:** brachiocephalic veins, central venous catheterization, central venous catheters, phlebography, pneumothorax

**Figure 1:**
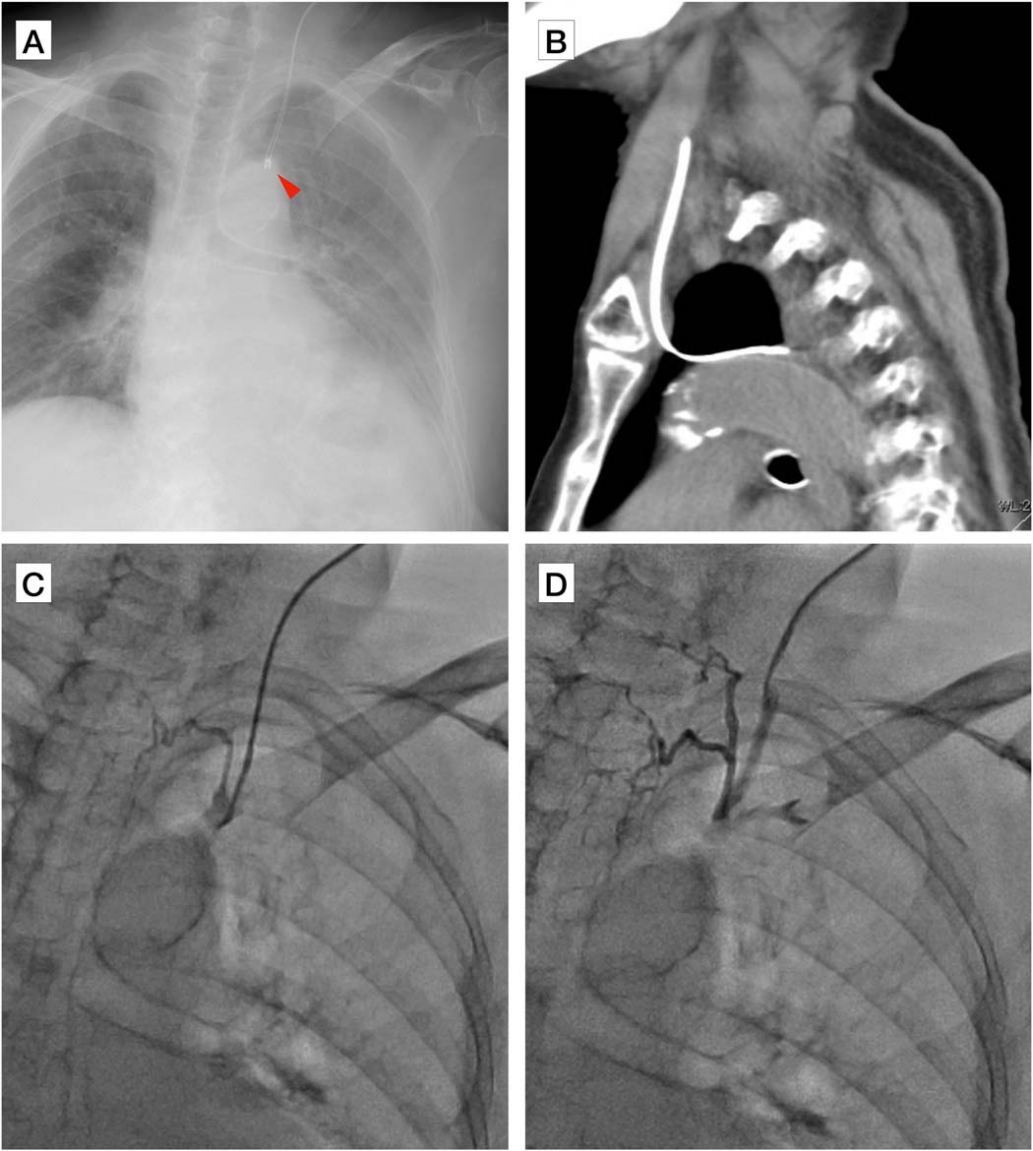
(**A**) Chest radiograph displaying dotted and displaced central venous catheter with hyperintense images (arrowhead). (**B**) Computed tomography scan showing the central venous catheter appearing to penetrate the pulmonary apex. (**C**) Venography through the central venous catheter showing catheter tip in the left superior intercostal vein. (**D**) Venography through the central venous catheter showing absence of the left brachiocephalic vein with catheter repositioned in the left internal jugular vein.

A 97-year-old female patient was admitted to our intensive care unit due to accidental hypothermia. A central venous catheter (CVC) was precisely inserted into the left internal jugular vein with successful aspiration. However, the tip of the CVC appeared displaced, dotted, and hyperintense on a chest radiograph (Fig. 1A). Computed tomography demonstrated that the CVC was inserted through the left internal jugular vein and appeared to penetrate the left pulmonary apex (Fig. 1B). Venography confirmed that the catheter tip was located in the left superior intercostal vein (Fig. 1C). Moreover, the left brachiocephalic vein was absent (Fig. 1D). The catheter was safely extracted, and a new CVC was inserted into the right internal jugular vein.

The catheter tip can be positioned in the left superior intercostal vein due to absence or occlusion of the left brachiocephalic vein, a rare anatomic anomaly [1]. Anatomic variations in left thoracic venous drainage, such as a persistent left superior vena cava, dominant flow of the superior intercostal vein into a hemiazygos vein, or a dilated superior intercostal vein, can easily result in mispositioning of the catheter tip [1]. Additional diagnostic procedures, such as venography, can exclude the occurrence of vascular perforation and confirm the presence of venous anatomical aberrations [2].
